# Effects of a Major Tree Invader on Urban Woodland Arthropods

**DOI:** 10.1371/journal.pone.0137723

**Published:** 2015-09-11

**Authors:** Sascha Buchholz, Hedwig Tietze, Ingo Kowarik, Jens Schirmel

**Affiliations:** 1 Department of Ecology, Technische Universität Berlin, Berlin, Germany; 2 Berlin-Brandenburg Institute of Advanced Biodiversity Research (BBIB), 14195 Berlin, Germany; 3 Institute for Environmental Sciences, University of Koblenz-Landau, Landau, Germany; Shandong University, CHINA

## Abstract

Biological invasions are a major threat to biodiversity; however, the degree of impact can vary depending on the ecosystem and taxa. Here, we test whether a top invader at a global scale, the tree *Robinia pseudoacacia* (black locust or false acacia), which is known to profoundly change site conditions, significantly affects urban animal diversity. As a first multi-taxon study of this kind, we analyzed the effects of *Robinia* dominance on 18 arthropod taxa by pairwise comparisons of woodlands in Berlin, Germany, that were dominated by *R*. *pseudoacacia* or the native pioneer tree *Betula pendula*. As a negative effect, abundances of five arthropod taxa decreased (Chilopoda, Formicidae, Diptera, Heteroptera, Hymenoptera); 13 others were not affected. Woodland type affected species composition of carabids and functional groups in spiders, but surprisingly did not decrease alpha and beta diversity of carabid and spider assemblages or the number of endangered species. Tree invasion thus did not induce biotic homogenization at the habitat scale. We detected no positive effects of alien dominance. Our results illustrate that invasions by a major tree invader can induce species turnover in ground-dwelling arthropods, but do not necessarily reduce arthropod species abundances or diversity and might thus contribute to the conservation of epigeal invertebrates in urban settings. Considering the context of invasion impacts thus helps to set priorities in managing biological invasions and can illustrate the potential of novel ecosystems to maintain urban biodiversity.

## Introduction

Biological invasions are a significant component of global change with a range of well documented adverse effects on biodiversity in invaded ecosystems [1, 2]. While our understanding of invasion impacts has clearly advanced in recent years [3–5], important questions should be considered in more detail in invasion impact studies [6]. These include (i) the direction of impacts [7, 8], i.e. whether the effects are positive, negative or missing, (ii) how impacts vary on temporal-spatial scales [9, 10], (iii) the extent to which the impacts depend on the type and range of taxa considered [7] and (iv) the relationship between human values and ecological effects in impact assessments [6, 11]. Impacts of the same invasive species can be multidirectional and vary among regions, ecosystems or taxa, as shown by studies on the invasive tree *Cinchona pubescens* [12, 13]. Multi-taxon studies that test for adverse biodiversity impacts (such as homogenization of species communities, decline of habitat specialists or endangered species) of a given invasive plant species at a regional scale can thus inform decisions on adequate management options and resource allocation.

Invasive tree species often form mono-specific stands and are major drivers of environmental change [14]. *Robinia pseudoacacia* (black locust or false acacia; henceforth *Robinia*) is native to southeast North America. Since its introduction to Europe in the first part of the 17^th^ century, it has been widely planted in Europe and Asia as an economically important multi-purpose tree [15]. It is now one of the top three invasive plant species in Europe [16, 17] and is also widespread in China [18] and Korea [19]. Due to its rapid growth, vigorous sprouting and capacity for symbiotic nitrogen fixation, *Robinia* can change ecosystem properties and plant species assemblages profoundly [15], e.g. by increasing total nitrogen, litter and organic carbon and decreasing total phosphorus [20–22]. As a consequence, *Robinia* is able to invade a range of ecosystem types in natural and urban settings, including nutrient-poor sites [15, 17, 23].

Invasions of dry grassland by *Robinia* usually conflict with conservation goals [15, 24] and are anticipated to increase with climate change [25]. A few studies have analyzed the impacts of *Robinia* on plant species in woodlands, and ambiguous results have been reported for plant diversity [24, 26–28]. Despite the significance of *Robinia* as an important tree invader, the effects the species has on animal taxa have clearly not yet been sufficiently studied [15]. It is thus an open question whether possible habitat alterations caused by *Robinia* translate into adverse impacts on animal diversity.

Plant invasions can have negative impacts on local faunal composition and biotic interactions [2, 29, 30], but positive effects on native animals have also been shown [8, 31, 32]. Since invertebrates represent the major component of biodiversity in terrestrial ecosystems [33], we analyzed the impacts of *Robinia* invasion on multiple arthropod taxa, with a particular focus on carabids (Coleoptera: Carabidae) and spiders (Araneae) that are useful ecological indicators in a broad variety of habitats [34]. An earlier study in urban grasslands suggests sensitive responses of both species groups to *Robinia* invasion [35].

To analyze impacts of *Robinia* invasion on multiple arthropod taxa, we used a study design with paired comparisons of pioneer woodlands on urban sites in Berlin, Germany, that were either dominated by the alien *Robinia* or by *Betula pendula* (henceforth *Betula*), a major native pioneer tree species [28]. The sampled stands result from succession on previously open urban sites and thus represent stages of forest recovery in urban settings. In face of rapid urbanization at the global scale, the importance of urban habitats for “biodiversity conservation where people live and work” [36] is increasing, and some urban habitats have been shown to function as habitat analogues of natural habitats [33, 37, 38].

While studies on urban habitat analogues mostly address open habitats, less attention has been paid to the role of invasive tree stands as a woodland analogue for, or threat to, animal conservation. A minor role of non-native tree species as habitat or food resource for native invertebrates is an important argument in conservation discussions [8], but has rarely been tested for invasive tree stands at the habitat scale. We anticipated significant invasion-mediated impacts on invertebrate diversity because *Robinia* is known to cause profound changes in habitat conditions and plant assemblages [20, 24, 28] that might affect related habitat or food resources for animals (e.g., more suitable habitat conditions for hydrophilic and shade-loving species, abundant detrital communities due to an increasing N).

The aim of this study is to analyze how the invasion by *Robinia* affects diversity patterns in multiple arthropod taxa of urban woodlands. In particular, the following research questions were addressed: (i) Do abundances of ground-dwelling and flying arthropods differ between native and non-native woodlands? (ii) Are there differences in diversity measures (species richness, Shannon diversity, evenness) and functional groups (indicated by ecological preference values for shade and moisture) of carabids and spiders between the two woodland types? (iii) Does the species composition of carabids and spiders differ between native and non-native woodlands? How do endangered species respond to tree invasion?

## Methods

### Study area and site selection

The study was carried out in the city of Berlin, Germany, with an area of 892 km² and about 3.5 million inhabitants. Berlin represents a complex urban matrix with a variety of land uses, in which woodlands amount to 21% [39]. In the past, urban woodlands developed mainly on derelict railway areas or formerly built-up areas that were destroyed during World War II or later emerged due to structural changes in the wake of the reunification of the city. The native *Betula pendula* and the alien *Robinia pseudoacacia* are the two most frequent pioneer trees on urban wastelands in Berlin, and both species establish dominant stands in the same type of habitats [40].

Using the study design of Trentanovi et al. ([28], see further details herein), we selected a set of ten pairs of urban woodlands (n = 20 sites) (S1 Appendix). Sites were open to the public. Our field studies did not involve any protected species. Thus no permits and approvals were required.

For each pair, one site was dominated by the native *Betula* (cover > 90%, *Robinia* absent; hereafter “native”) and the other by the non-native *Robinia* (cover > 90%, *Betula* absent; hereafter “non-native”). We used a paired design to control for possible co-factors. The study design implies that the two sites of a pair were approximately of the same age, with a maximum age of about 60 years for the post-WW II stands, belonged to the same habitat type (e.g., pioneer forest, pre-forest according to the biotope map of Berlin [39]), and had the same land-use history and the same type of soil [28]. Plant species richness and composition clearly differed between native and non-native woodland patches [28]. Previous studies have shown that dominance of *Betula* or *Robinia* in urban woods in Berlin does not reflect differences in soil conditions prior to tree establishment but depends on which species initially established in the course of succession [41]. The minimum size of sites was 30 m² and we kept a minimum distance of 20 m between both sites of one pair to preclude neighbouring effects. We also kept a distance of 20 m from the border of the woodland patch. All pairs were separated by a minimum distance of 1 km. To test for effects of the surrounding land-use types we analyzed the surrounding urban matrix configuration (e.g. proportion of forests or impervious surfaces) within a 100 m radius around the centre of each site using GLMM (S2 and S3 Appendixes).

### Sampling of arthropods and environmental parameters

We sampled ground-dwelling arthropods using pitfall traps. We installed three uncovered pitfall traps at random locations on each site but keeping a minimum distance of 5 m between traps. Traps were 500 ml plastic cups (9 cm diameter, 12 cm depth) one-fourth filled with a 4% formalin-detergent solution. Sampling was done from 1 May to 30 June 2012, and traps were controlled every two weeks but emptied every four weeks. Although a two month sampling period is rather short, recent studies have shown that this period is sufficient to yield reliable data [42; 43]. Three pitfall traps per site are considered as the minimum number required to obtain reliable data [44]. All arthropods were removed, identified as Araneae, Carabidae, Chilopoda, Collembola, Dermaptera, Diplopoda, Formicidae, Isopoda, Opiliones or Staphylinidae and then transferred to 75% ethanol. We identified carabid beetles and spiders to species level using standard determination keys [45–47].

Flying insects were caught using glue traps. At each site, we attached three traps to branches at a height of 2 metres. The glue traps consisted of transparent plastic sheets (12 × 12 cm) covered with sticky glue on one side (SOVEU-RODE aerosol, Witasek, Feldkirchen/Kärnten, Austria). Traps were open from 1 May to 30 June 2012 and also were controlled every two weeks for functionality. We counted flying insects of the following orders: Auchenorrhyncha, Coleoptera, Diptera, Heteroptera, Hymenoptera, Lepidoptera, Neuroptera, Thysanoptera.

For each site we recorded environmental parameters that might underlie patterns of arthropod diversity at the site or landscape scale. The proportions of bare ground, litter cover, moss cover, and herb cover were estimated for an area of 1 m² around each pitfall trap and averaged per site for statistical analysis. Furthermore, we estimated shrub cover and canopy cover at site level using a 10 m² reference area. Moreover, we calculated the proportion of forested area, open habitat, water, garden and impervious area within a radius of 100 m of the centre of each site (S2 Appendix).

### Data analysis

All pitfall traps and glue traps per site were treated as a sample unit. For pitfall trap data, activity densities of ground-dwelling arthropods were expressed as the raw individual numbers. Because some glue traps were lost or damaged, abundances of flying arthropods were the standardized number of individuals (individuals/trap/site).

To analyze differences in ecological preferences among carabid and spider species assemblages, we used shade and moisture preference values from Irmler and Gürlich [48] for carabids, and from Entling et al. [49] for spiders. For carabids, values for shading range from 0 to 14. Shading value 0 is for those carabid species which prefer the most open habitats and 14 is for those species which prefer the most shaded habitats (forests). Carabid moisture values range from 0 (most xerophilic species) to 9 (most hydrophilic species). For spiders, niche position values range on a continuous scale from 0 to 1. Niche position shading is 0 for the species which prefer the most open habitats and 1 for those which prefer the most shaded habitats (forests). Niche position for moisture is 0 for species which prefer the moistest habitats and 1 for those which prefer the driest habitats. Status of endangerment was assessed according to the Red List of Berlin [50, 51].

We used random effect generalized models (GLMM) for paired comparisons with woodland type (native/non-native) as a fixed effect and location (pairing factor of the sites) as a random effect (command ‘glmmPQL’ in R-package ‘MASS’ [52]). Activity densities of ground-dwelling arthropods and the combined number of endangered carabid and spider species were tested with Poisson GLMMs for count data. Abundances of flying arthropods as well as diversity measures (species richness, Shannon diversity, Shannon Evenness) and ecological preference values of carabids and spiders were tested with Gaussian GLMMs on log10(x+1) transformed data to meet assumptions. Where significant differences were observed between native and non-native sites, we used GLMM with vegetation parameters as fixed effects and location as a random effect. As predictor variables the cover of mosses, herbs, shrubs, and canopy were included in the full models; due to collinearity (Pearson´s r > |0.7|), litter cover (with herbs) and the proportion of bare ground (with canopy) were not included. For model selection non-significant predictor variables were excluded stepwise from the models. Significance of *p*-values was based on χ²-statistics [53]. Differences in vegetation parameters were also tested with Gaussian GLMMs, and data was log10(x+1) transformed if model assumptions were violated. Model performance was checked graphically using diagnostic plots [53].

Differences in species compositions of carabids and spiders between the two woodland types were analyzed with a permutational multivariate ANOVA (command ‘adonis’ in R package ‘vegan’ [54]). Location was again used as pairing factor of the sites (strata = location). Rare species (less than four individuals) were deleted to reduce the statistical noise in the data set. We used the Bray-Curtis distance as distance measure. Significance was tested with permutation tests (9999 permutations) with pseudo-F ratios. To test whether the species compositions of both woodland types differ in their variances (beta diversity), we analyzed the multivariate dispersion based on the Bray-Curtis similarity of species (using the command ‘betadisper’ in the R package ‘vegan’). A permutational ANOVA was used to test for differences between the multivariate homogeneity of dispersions of native and non-native sites. All statistical analyses were done using the free software environment R 2.11.1 [55].

## Results

### Environmental parameters

Herb cover was significantly higher in non-native stands than in native stands (Table 1). All other vegetation parameters did not differ between woodland types. Moreover, there were no differences in the urban matrix composition in the surroundings of native and non-native stands (S2 Appendix), and urban matrix parameters had no significant impact on the investigated taxa. In contrast, environmental parameters significantly influenced species activity densities of some arthropod taxa (Table 2). Herbal layer negatively affected Chilopoda (*t* = -3.047, *p* = 0.014), Formicidae (*t* = -2.632, *p* = 0.008) and Diptera (*t* = -4.166, *p* = 0.004). Mosses had a positive effect on ants (*t* = 3.519, *p* = 0.008), while canopy density negatively influenced Heteroptera (*t* = -6.362, *p* < 0.001) but positively affected the shading preference of spiders (*t* = 3.530, *p* = 0.008).

**Table 1:** Vegetation characteristics of native (*Betula pendula*) and non-native (*Robinia pseudoacacia*) urban woodland pairs (*n* = 10). Differences were tested with Gaussian GLMMs. Significant results at *p* < 0.05 are shown in bold.

Cover/proportion (%)	Native	Non-native	*t*	*p*
Bare ground	16.0 ± 7.4	6.0 ± 3.9	1.581	0.651^#^
Litter	54.5 ± 11.1	35.0 ± 11.3	-1.230	0.250
Mosses	4.5 ± 2.0	3.0 ± 1.5	-0.896	0.615^#^
Herbs	33.0 ± 10.6	71.0 ± 9.8	3.024	**0.014**
Shrubs	12.5 ± 3.7	21.5 ± 5.4	1.711	0.097^#^
Canopy	69.0 ± 6.6	81.0 ± 4.9	1.494	0.170

^#^ data log10(x+1) transformed

**Table 2:** Effect of vegetation parameters on abundances of arthropods and the shading preference values of spiders in native and non-native urban woodlands. Only taxa/ecological preference values were analyzed which differed between native and non-native woodlands (Tables 3 and 4). Differences were tested with GLMMs. Significant results at *p* < 0.05 are shown in bold.

Response	Predictors	Estimate	SE	*t*	*p*
Chilopoda	Herbs	–0.018	0.006	–3.047	**0.014**
Formicidae	Mosses	0.077	0.022	3.519	**0.008**
	Herbs	–0.011	0.004	–2.632	**0.030**
Diptera	Herbs	–0.004	0.001	–4.166	**0.004**
Heteroptera	Canopy	–0.010	0.002	–6.362	**<0.001**
Shading (Araneae)	Canopy	0.000	0.000	3.530	**0.008**

### Abundances of ground-dwelling and flying arthropods

Out of 18 arthropod taxa, five showed higher abundances in native than in non-native woodlands, while no taxon was more abundant in non-native woodlands (Table 3). For ground-dwelling arthropods, Chilopoda (*t* = -2.286, *p* = 0.048) and Formicidae (*t* = -4.534, *p* = 0.001) were more abundant in native woodlands, while no significant differences between both woodland types were found for Araneae, Carabidae, Collembola, Dermaptera, Diplopoda, Isopoda, Opiliones, and Staphylinidae. For flying arthropods, significantly higher abundances were detected in native stands for Diptera (*t* = -2.733, *p* = 0.026), Heteroptera (*t* = -2.599, *p* = 0.032) and Hymenoptera (*t* = -2.794, *p* = 0.023). No significant differences were found between the two woodland types for Auchenorrhyncha, Coleoptera, Lepidoptera, Neuroptera and Thysanoptera.

**Table 3:** Abundances (mean ± standard error) of ground-dwelling and flying arthropods in native (*Betula pendula*) and non-native (*Robinia pseudoacacia*) woodlands. Differences for ground-dwelling arthropods were tested with Poisson GLMM for count data and for flying arthropods with Gaussian GLMMs on log10(x+1) transformed data. Significant results at *p* < 0.05 are shown in bold.

Response	Native	Non-native	*t*	*p*
Ground-dwelling arthropods				
Araneae	278 ± 50	317 ± 73	0.444	0.668
Carabidae	48 ± 20	31 ± 6	-1.348	0.211
Chilopoda	5 ± 1	2 ± 1	-2.286	**0.048**
Collembola	48 ± 24	81 ± 54	0.955	0.365
Dermaptera	4 ± 1	9 ± 3	2.127	0.062
Diplopoda	387 ± 272	274 ± 75	-0.855	0.415
Formicidae	226 ± 21	83 ± 28	-4.534	**0.001**
Isopoda	188 ± 86	199 ± 77	0.275	0.789
Opiliones	18 ± 6	9 ± 4	-1.242	0.246
Staphylinidae	16 ± 5	16 ± 4	-0.097	0.925
Flying arthropods				
Auchenorrhyncha	6 ± 2	8 ± 3	0.356	0.731
Coleoptera	8 ± 1	9 ± 2	0.053	0.959
Diptera	216 ± 24	135 ± 16	-2.733	**0.026**
Heteroptera	2 ± 0	0 ± 0	-2.599	**0.032**
Hymenoptera	63 ± 8	37 ± 5	-2.794	**0.023**
Lepidoptera	1 ± 0	1 ± 0	-0.831	0.433
Neuroptera	1 ± 0	0 ± 0	-0.453	0.663
Thysanoptera	8 ± 2	14 ± 5	1.230	0.253

### Alpha diversity and ecological preference values of carabids and spiders

A total of 785 individuals from 53 native carabid beetle species were recorded (S4 Appendix). Of these, *Nebria brevicollis* (*n* = 239) was most abundant, followed by *Carabus nemoralis* (*n* = 57) and *Amara ovata* (*n* = 51). A total of 5,612 spider individuals belonging to 100 native species were caught. The most frequent species by far was *Pardosa lugubris* (*n* = 3,632), followed by *Ozyptila praticola* (*n* = 603), *Tenuiphantes flavipes* (*n* = 313) and *Euryopis flavomaculata* (*n* = 103).

No differences were found in alpha diversity measures (species richness, Shannon diversity, evenness) for either carabids or spiders between native and non-native woodlands (Table 4). The shade preference value of spiders was significantly lower in native than in non-native woodlands (*t* = 2.749, *p* = 0.023) and was positively correlated with canopy density (Table 2). In contrast, the moisture preference value of spiders did not differ significantly (Table 4). For carabids, no differences in ecological preference values were detected (Table 4).

**Table 4:** Measures of alpha diversity and ecological indicator values for shading and moisture (mean ± standard error) of carabid beetles and spiders in native (*Betula pendula*) and non-native (*Robinia pseudoacacia*) woodlands. Differences were tested with Gaussian GLMMs on log10(x+1) transformed data. Significant results at *p* < 0.05 are shown in bold.

Response	Native	Non-native	*t*	*p*
Carabidae				
Species richness	9 ± 2	9 ± 1	-0.048	0.963
Shannon diversity	1.54 ± 0.11	1.76 ± 0.13	1.308	0.223
Evenness	0.67 ± 0.09	0.72 ± 0.04	0.499	0.629
Shading	1.56 ± 0.39	1.61 ± 0.28	0.110	0.915
Moisture	3.22 ± 0.37	2.65 ± 0.16	-1.479	0.173
Araneae				
Species richness	21 ± 3	19 ± 2	-0.727	0.486
Shannon diversity	1.46 ± 0.22	1.46 ± 0.18	-0.010	0.992
Evenness	0.26 ± 0.04	0.26 ± 0.03	0.499	0.629
Shading	0.40 ± 0.01	0.44 ± 0.01	2.749	**0.023**
Moisture	0.42 ± 0.01	0.40 ± 0.01	-1.972	0.080

### Species composition of carabids and spiders

The species composition of carabids was significantly related to woodland type (F = 2.369, R² = 0.12, *p* = 0.009). In contrast, woodland type had no significant effect on spider species composition (F = 1.054, R² = 0.05, *p* = 0.238).

**Fig 1:**
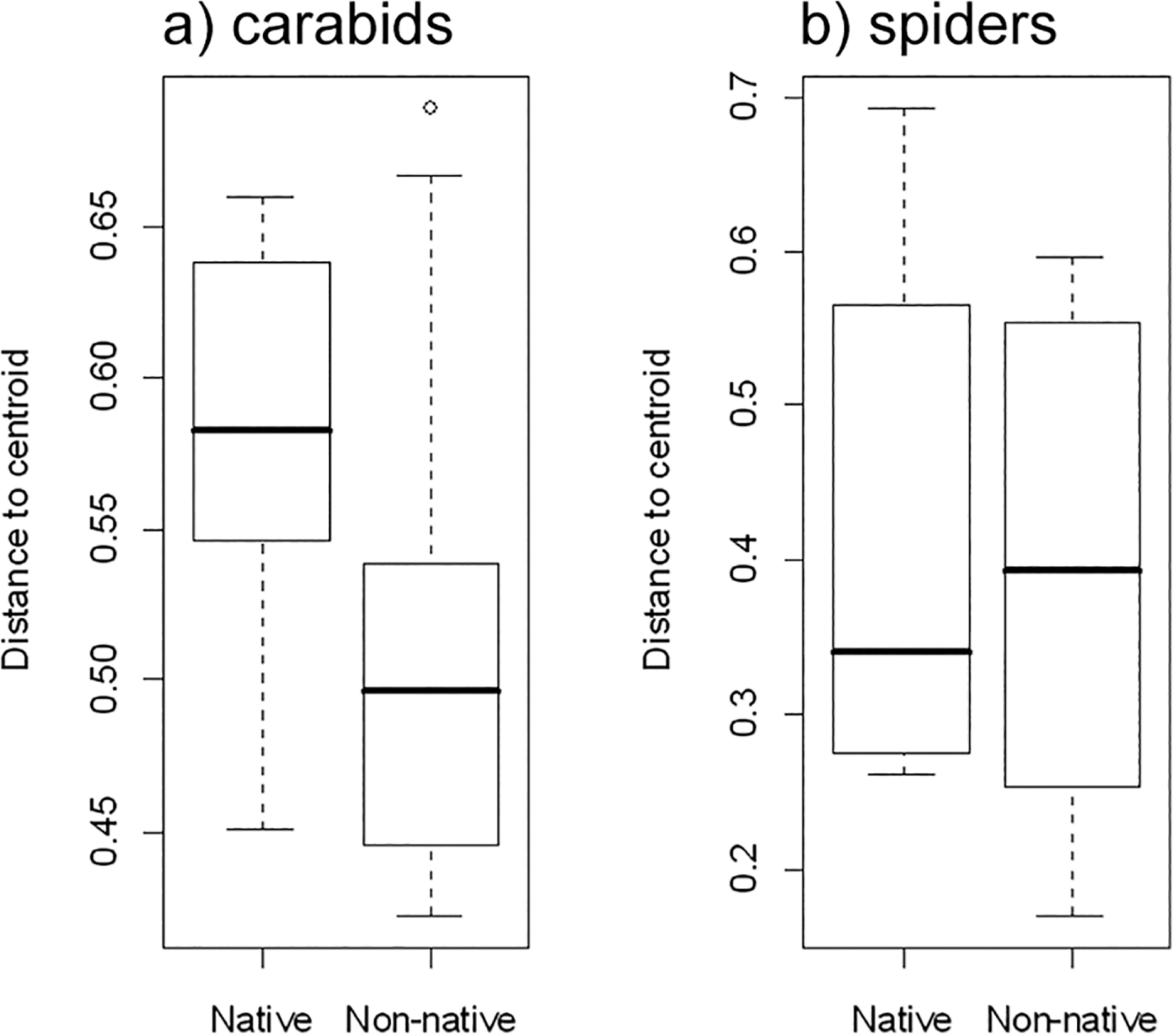
Variances in species composition (beta diversity) for a) carabids and b) spiders of native (*Betula pendula*) and non-native (*Robinia pseudoacacia*) woodlands. To test whether variances (multivariate dispersion) differed between the two woodland types, the distances of group members to the group centroid were subjected to ANOVA (carabids: *F* = 3.164, *p* = 0.092; spiders: *F* = 0.082, *p* = 0.779).

**Fig 2:**
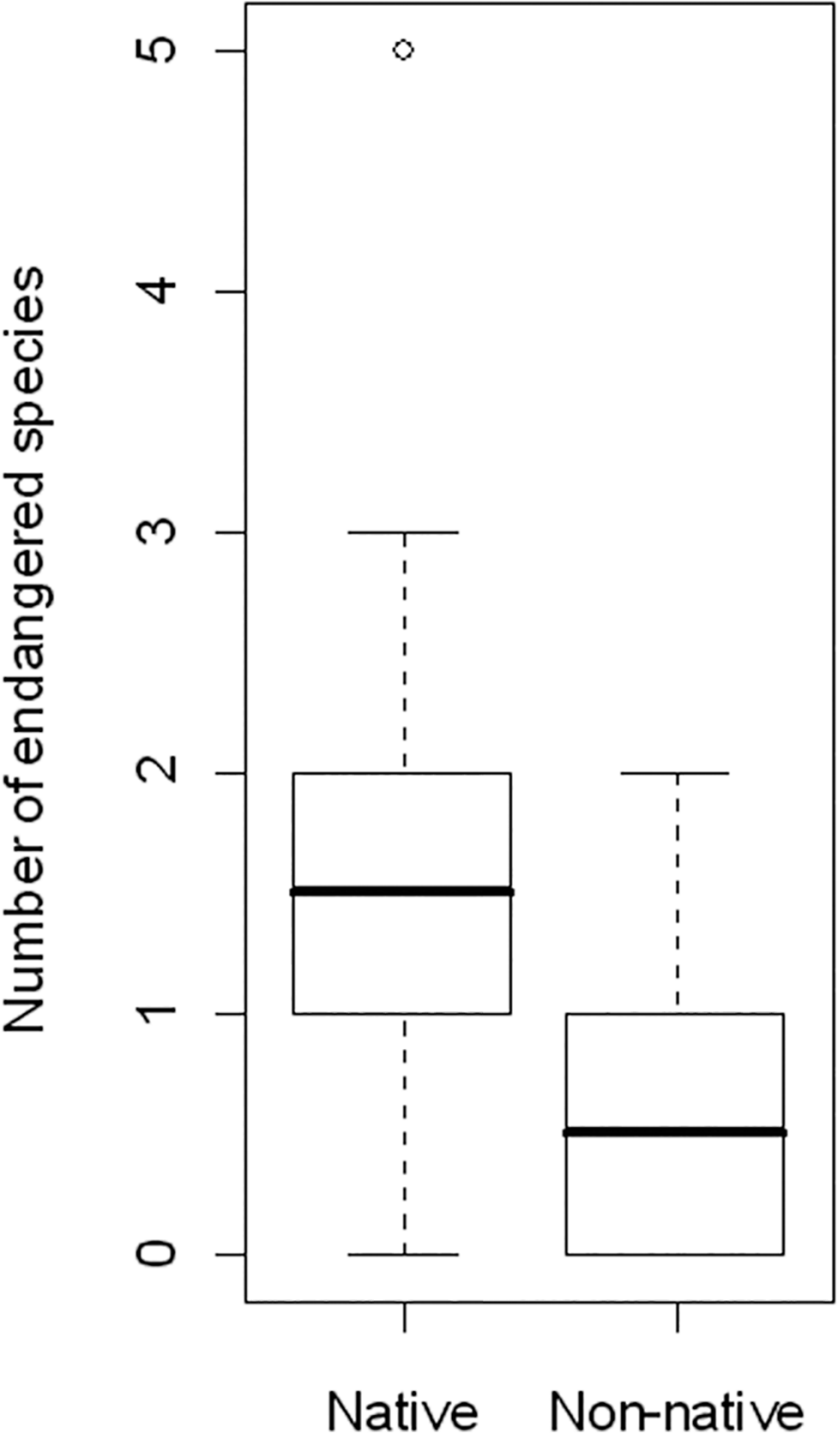
Combined number of endangered carabid [51] and spider [50] species in native (*Betula pendula*) and non-native (*Robinia pseudoacacia*) woodlands. Differences were not significant (GLMM: *t* = -2.092, *p* = 0.066).

Variances in species composition (beta diversity) did not differ significantly between the two woodland types (Fig 1A and 1B) for either carabids or spiders, nor did the combined numbers of endangered carabid and spider species (Fig 2).

## Discussion

Comparing invaded sites to nearby non-invaded sites is a well-established approach in invasion ecology to assess invasion impacts when data on temporal changes are lacking [56, 57], although results might be affected by pre-invasion differences between sites [58]. To minimize such effects, we located the compared pairs of native and non-native woodlands in the same environmental context (same habitat type, land-use history, no differences in the surrounding urban matrix). Differences in parameters of stand structure were also lacking, with the exception of herb cover, which was higher under *Robinia* (Table 1). Yet the latter is a well-known invasion effect due to the enhanced nitrogen availability under the *Robinia* canopy [15]. We thus expect that our study was able to reveal effects of native versus non-native tree-dominance on arthropod diversity in urban woodlands.

Applying a multi-taxon approach showed impacts of a dominant alien tree species, compared to a dominant native tree species, on invertebrate taxa in urban woodlands. Due to changes in the herb cover (Table 1) and the plant species composition in the wake of *Robinia* invasion [28] we expected clear differences in arthropod diversity patterns between native and non-native woodlands. Indeed, woodland type was related to species composition of carabids and functional groups (moisture preference) in spiders, which suggests an invasion-mediated species turn-over in these animal groups. We detected no positive effects of the alien tree species, but negative effects in five out of 18 arthropod taxa in terms of decreased abundances (Table 3). Alpha diversity, variations in species composition (beta diversity), and the number of endangered species of carabids and spiders were not related to woodland type. Thus, the second main result of our study showed that significant relationships between woodland type and abundances of arthropod taxa and diversity of carabid and spider assemblages were largely missing. These results were counterintuitive because carabids and spiders have been shown to respond sensitively to plant invasions [30, 59–61].

### Abundances of ground-dwelling and flying arthropods

Only Chilopoda and Formicidae were more abundant in native than in non-native woodlands. This was mainly due to the more open herbal layer in native birch woodlands (Table 2). In central Europe, Formicidae species are mostly xerophilic [62] and thus may avoid the dense understory vegetation in *Robinia* stands that is facilitated by enhanced nitrogen availability [15]. Accordingly, herbal layer significantly decreased ant activity densities (Table 2). However, pitfall trap data for Formicidae is difficult to explain, because catch numbers often do not represent activity densities but rather depend on the colony size and behavior (e.g. clustered occurrence near nests and trails [63, 64]. Furthermore, ants are social insects and thus the individual is not a reproductive unit [65]. Chilopoda are mostly generalist predators of the litter layer. Our results did not indicate higher prey availability in native woodlands (e.g. Collembola, Isopoda; Table 3); however, important food sources such as Dipteran larvae or Lumbricidae were not analyzed. As for ants, Chilopoda were negatively affected by a denser herbal layer (Table 2) and it might be that accessibility of litter layer is reduced in denser vegetation.

In the case of flying arthropods, the orders Diptera, Heteroptera, and Hymenoptera had higher abundances in native woodlands. Diptera were related to the more open herbal layer of native woodlands, while Heteroptera (and as a trend Hymenoptera) were related to a more open canopy layer (Table 2). Beside these preferences for different vegetation structures, all three orders contain several herbivore or pollinator species, which often prefer native plants to exotic ones [66, 67]. The sampled *Betula* stands are richer in native plant species compared to the *Robinia* stands [28] and thus likely offer more food resources. Moreover, *Betula* as a dominant tree is much more attractive for herbivores than *Robinia* [68]. Lower herbivore pressure in Europe compared to the native North American range [15] is assumed to be a contributory factor in the successful spreading and the longer persistence of *Robinia* during forest succession in the invaded range [40]. *Robinia* shows a higher investment into reproductive organs compared to native trees [69]. In consequence, large flower crops of *Robinia* provides abundant nectar resources; in Europe, however, honeybees are the main pollinators, and the role of floral visits by other insects remains unclear to date [15]. *Robinia* is thus likely attractive mainly for larger pollinators such as bees, which are able to depress the keel and wing petals of the flower in order to reach the nectar. Correspondingly, abundances of Hymenoptera species–including several pollinator species–were higher in *Betula* than in *Robinia* woodlands in our study. However, as we focussed on abundances of flying arthropods, further qualitative studies might find differences in species and trait compositions of arthropod taxa related to *R*. *pseudoacacia* invasion.

### Carabids and spiders

Carabids and spiders are known to be very sensitive to environmental modifications even at small scales [70, 71]. Despite clear structural differences in the cover of the herb layer between native and non-native stands, significant impacts of *Robinia* invasion on alpha diversity were not apparent. This is in line with Sax [56], who found no differences in species diversity across multiple taxonomic groups (understory plants, leaf-litter invertebrates, amphibians and birds) between woodlands dominated by other native and non-native woody species. Brown et al. [72] also argued that species richness will often remain relatively stable regardless of whether dominant vegetation type or plant species composition changes. Yet comparisons of plant species richness of *Robinia* stands with stands of native woody species illustrate positive [24], negative [28] as well as missing [26] significant effects of the tree invader. These findings clearly indicate limits of generalizations on invasion impacts of a given species and illustrate the dependence of impacts on the study system and the group(s) of taxa considered.

Exotic species can contribute to biotic homogenization as mostly discussed for larger spatial scales [73, 74]. We could not detect any difference in the variation in carabid and spider species compositions (beta diversity) between native and non-native woodlands. *Robinia* as a dominant tree invader therefore did not homogenize carabid and spider assemblages of urban woodlands, and non-native woodlands showed a similar community differentiation to native woodlands. This confirms similar results on beta diversity of plant assemblages in *Robinia* versus *Betula* stands [28].

Both urban woodland types provided suitable habitats for some endangered carabid and spider species (N = 14 in total), but the combined number of endangered species did not statistically differ between them (Fig 2). In contrast to alpha and beta diversity, the species composition of carabids differed between the two woodland types, likely reflecting changes in habitat structure. For spiders, the number of species preferring shady conditions was higher in non-native stands, which correlates with a higher canopy density in non-native woodlands (Table 2). However, differences were rather low (a shift from 0.40 to 0.44, see Table 4) so biological significance should not be overestimated.

### Implications for urban conservation approaches

While there is broad evidence of adverse biodiversity effects driven by *Robinia* invasions in open ecosystems which clearly conflict with conservation goals [15], our study demonstrates minor invasion-mediated effects on arthropod taxa in urban pioneer woodlands, which was especially true for carabids and spiders. We found no significant decrease in biodiversity or occurrences of endangered species, which suggests that non-native *Robinia* woodlands may function as a habitat analogue for ground-dwelling invertebrates. *Robinia* stands might therefore contribute to an extent similar to that of native pioneer woodland to carabid and spider species conservation in urban settings. While at the species scale, *Robinia* is much less attractive for invertebrates than its native counterpart *Betula* [68], our results suggest that such differences do not necessarily translate to the habitat scale in the case of urban pioneer woodlands. In consequence, replacing urban *Robinia* stands by stands dominated by native *Betula* would not be justified in terms of epigeal invertebrate conservation. Considering the context dependence of invasion impacts thus helps to set priorities in managing biological invasions at the regional scale.

Differences between native and non-native stands might emerge in the future due to temporal invasion dynamics [9]. Yet the dominance of *Robinia* is expected to decrease in the long run due to competition by shade-tolerant native tree species [40]. Thus, an intriguing field of long-term research will be to test for the reliability of our present results in relation to evolving forest systems. Moreover, the integration of specific species, trophic groups, species traits and further landscape factors into future research would help to reveal possible invasion impacts that our methodological approach was unable to detect. As spiders and carabids are generalist predators, further studies should test whether pronounced effects might be seen in more specialized taxa (e.g., parasitoids, chrysomelids, Lepidoptera) and canopy arthropod species.

We clearly demonstrated invasion-mediated changes in carabid species composition. These may be assessed as negative, neutral or positive, depending on the referenced value system, because changes in ecosystem characteristics need not necessarily be assessed as adverse impacts or ecological damage [11, 75]. It might be a conservation goal to restore native forest vegetation by controlling *Robinia* and enhancing native tree species. Due to the vigorous vegetative regeneration of this tree [15], however, such measures and the necessary maintenance over the following years would be cost intensive and likely increase CO_2_ emissions [76]–in contrast to spontaneous forest succession. This is an advantage of wild-grown woods in terms of ecosystem services.

As the sampled pioneer forests on urban ground represent novel ecosystems that reflect an adaptation to profoundly changed urban sites, we might also accept changes in species assemblages of such settings [38]. Stands of alien tree species could thus be accepted on urban sites, given that invasions of susceptible habitats with a high conservation value can be excluded or easily managed. In Berlin, urban wastelands with a co-occurrence of native and non-native woodlands could thus integrated into the urban green infrastructure, including legally protected conservation areas, without any efforts to reduce the dominance of non-native tree species within pioneer forests [41]. The main idea hereby was to allow ecosystem processing without human interventions that aim to develop forests towards historical benchmarks. Invasions of adjacent grassland by *Robinia* are, however, being controlled. Beyond functioning as habitat analogues for native species, novel urban woodlands may provide manifold opportunities for experience of nature and can help to link urban dwellers with nature [36].

## Supporting Information

S1 Appendix Geographic coordinates of field study sites (N = north, E = east). (DOC)

S2 Appendix Comparison of the urban matrix composition between native and non-native woodland site pairs. The proportion of forest, open habitat, impervious surface, garden, and water was calculated with GIS for a 100 m radius of each site. Differences were tested with GLMM.
(DOC)

S3 Appendix Site data. (DOC)

S4 Appendix Species list and individual numbers (total sum and mean ± sem) of carabids and spiders in native (*Betula pendula*) and non-native (*Robinia pseudoacacia*) urban woodlands. Differences were tested with Poisson GLMM for count data. Only species with ≥ 20 individuals were tested. Explanations: * = endangered species according to the Red List of Berlin [50, 51].
(DOC)
