# Supplementary material for: Effects of a Major Tree Invader on Urban Woodland Arthropods
Source: PLoS One. 2015 Sep 11;10(9):e0137723. doi: 10.1371/journal.pone.0137723 (PMC4567378; doi:10.1371/journal.pone.0137723)
Supplement: S1 Appendix — (DOC) [file pone.0137723.s001.doc]

**S1 Appendix.** Geographic coordinates of field study sites (N = north, E = east).

| **No.** | **N** | **E** |
| --- | --- | --- |
| **Native birch** | | |
| 1.1 | 52°29’28.72’’ | 13°22’31.67’’ |
| 2.1 | 52°29’26.84’’ | 13°22’27.60’’ |
| 3.1 | 52°24’17.97’’ | 13°18’25.66’’ |
| 4.1 | 52°24’22.72’’ | 13°18’28.55’’ |
| 5.1 | 52°24’25.81’’ | 13°18’35.51’’ |
| 6.1 | 52°24’19.22’’ | 13°23’11.05’’ |
| 7.1 | 52°24’21.40’’ | 13°23’08.50’’ |
| 8.1 | 52°29’19.70’’ | 13°14’37.07’’ |
| 9.1 | 52°29’20.53’’ | 13°14’32.99’’ |
| 10.1 | 52°27’41.12’’ | 13°21’33.10’’ |
| **Non-native black locust** | | |
| 1.2 | 52°29’22.71’’ | 13°22’25.07’’ |
| 2.2 | 52°29’29.25’’ | 13°22’24.65’’ |
| 3.2 | 52°24’17.55’’ | 13°18’27.90’’ |
| 4.2 | 52°24’23.60’’ | 13°18’30.53’’ |
| 5.2 | 52°24’25.38’’ | 13°18’33.34’’ |
| 6.2 | 52°24’13.48’’ | 13°23’11.99’’ |
| 7.2 | 52°24’16.92’’ | 13°23’11.69’’ |
| 8.2 | 52°29’17.38’’ | 13°14’39.35’’ |
| 9.2 | 52°29’14.83’’ | 13°14’34.43’’ |
| 10.2 | 52°27’43.95’’ | 13°21’34.13’’ |
