# Supplementary material for: Effects of a Major Tree Invader on Urban Woodland Arthropods
Source: PLoS One. 2015 Sep 11;10(9):e0137723. doi: 10.1371/journal.pone.0137723 (PMC4567378; doi:10.1371/journal.pone.0137723)
Supplement: S2 Appendix — The proportion of forest, open habitat, impervious surface, garden, and water was calculated with GIS for a 100 m radius of each site. Differences were tested with GLMM. (DOC) [file pone.0137723.s002.doc]

**S2 Appendix.** Comparison of the urban matrix composition between native and non-native woodland site pairs. The proportion of forest, open habitat, impervious surface, garden, and water was calculated with GIS for a 100 m radius of each site. Differences were tested with GLMM.

| **Cover/proportion (%)** | **Native** | **Non-native** | ***t*** | ***p*** |
| --- | --- | --- | --- | --- |
| Forest | 54.0 ± 5.6 | 57.0 ± 5.0 | 0.624 | 0.548 |
| Open habitat | 25.6 ± 5.5 | 25.2 ± 3.8 | -0.090 | 0.931 |
| Impervious surface | 17.8 ± 4.7 | 16.9 ± 5.6 | -0.721 | 0.489 |
| Garden | 1.6 ± 0.9 | 0.4 ± 0.3 | -1.324 | 0.218 |
| Water | 0.5 ± 0.3 | 0.5 ± 0.5 | -0.572 | 0.582 |
