# Supplementary material for: Effects of a Major Tree Invader on Urban Woodland Arthropods
Source: PLoS One. 2015 Sep 11;10(9):e0137723. doi: 10.1371/journal.pone.0137723 (PMC4567378; doi:10.1371/journal.pone.0137723)
Supplement: S3 Appendix — (DOC) [file pone.0137723.s003.doc]

**S3 Appendix.** Site data

| **pair** | **tree species** | **canopy** | **shrub** | **herb** | **litter** | **moss** | **forest area** | **open habitat** | **water** | **garden** | **impervious** |
| --- | --- | --- | --- | --- | --- | --- | --- | --- | --- | --- | --- |
| 1 | black locust | 95 | 5 | 95 | 5 | 0 | 57 | 24 | 0 | 0 | 19 |
|  | birch | 90 | 5 | 5 | 90 | 0 | 50 | 22 | 0 | 0 | 28 |
| 2 | black locust | 90 | 10 | 80 | 15 | 0 | 51 | 18 | 0 | 3 | 28 |
|  | birch | 95 | 20 | 0 | 90 | 5 | 46 | 14 | 0 | 2 | 38 |
| 3 | black locust | 90 | 15 | 100 | 0 | 0 | 29 | 28 | 0 | 0 | 43 |
|  | birch | 70 | 20 | 70 | 50 | 5 | 31 | 37 | 0 | 0 | 31 |
| 4 | black locust | 85 | 15 | 80 | 70 | 0 | 50 | 0 | 0 | 1 | 48 |
|  | birch | 60 | 5 | 80 | 50 | 5 | 57 | 0 | 0 | 8 | 34 |
| 5 | black locust | 95 | 40 | 0 | 90 | 0 | 36 | 44 | 5 | 0 | 15 |
|  | birch | 40 | 40 | 20 | 10 | 0 | 42 | 34 | 3 | 0 | 21 |
| 6 | black locust | 80 | 30 | 95 | 0 | 15 | 76 | 23 | 0 | 0 | 1 |
|  | birch | 40 | 0 | 80 | 5 | 20 | 50 | 39 | 0 | 0 | 10 |
| 7 | black locust | 50 | 10 | 50 | 80 | 0 | 65 | 35 | 0 | 0 | 0 |
|  | birch | 50 | 5 | 50 | 10 | 10 | 93 | 6 | 0 | 0 | 0 |
| 8 | black locust | 60 | 10 | 95 | 0 | 5 | 78 | 22 | 0 | 0 | 0 |
|  | birch | 85 | 5 | 5 | 90 | 0 | 73 | 25 | 2 | 0 | 0 |
| 9 | black locust | 75 | 20 | 65 | 40 | 5 | 62 | 37 | 0 | 0 | 1 |
|  | birch | 90 | 10 | 20 | 80 | 0 | 41 | 59 | 0 | 0 | 0 |
| 10 | black locust | 95 | 60 | 50 | 50 | 5 | 66 | 21 | 0 | 0 | 14 |
|  | birch | 70 | 15 | 0 | 70 | 0 | 57 | 20 | 0 | 6 | 16 |
