# Supplementary material for: Effects of a Major Tree Invader on Urban Woodland Arthropods
Source: PLoS One. 2015 Sep 11;10(9):e0137723. doi: 10.1371/journal.pone.0137723 (PMC4567378; doi:10.1371/journal.pone.0137723)
Supplement: S4 Appendix — Differences were tested with Poisson GLMM for count data. Only species with ≥ 20 individuals were tested. Explanations: * = endangered species according to the Red List of Berlin [50, 51]. (DOC) [file pone.0137723.s004.doc]

**S4 Appendix.** Species list and individual numbers (total sum and mean ± sem) of carabids and spiders in native (*Betula pendula*) and non-native (*Robinia pseudoacacia*) urban woodlands. Differences were tested with Poisson GLMM for count data. Only species with ≥ 20 individuals were tested. Explanations: * = endangered species according to the Red List of Berlin [50, 51].

|  | **native** |  | **non-native** | | ***t*** | ***p*** |
| --- | --- | --- | --- | --- | --- | --- |
|  | sum | mean ± SE | sum | mean ± SE |  |  |
| **Carabids** |  |  |  |  |  |  |
| *Amara communis* | 38 | 4 ± 2 | 6 | 1 ± 0 | -3.631 | 0.006 |
| *Amara consularis* | 9 | . | 9 | . |  |  |
| *Amara familiaris* | 1 | . | 0 | . |  |  |
| *Amara ovata* | 0 | 0 ± 0 | 51 | 5 ± 2 | 0.999 | 0.001 |
| *Amara similata* | 0 | 0 ± 0 | 36 | 4 ± 2 | 9.452 | < 0.001 |
| *Anisodactylus binotatus* | 1 | . | 0 | . |  |  |
| *Asaphidion flavipes* | 0 | . | 5 | . |  |  |
| *Anchomenus dorsalis* | 0 | . | 1 | . |  |  |
| *Badister bullatus* | 7 | 1 ± 0 | 16 | 2 ± 1 | 1.400 | 0.195 |
| *Badister sodalis* | 2 | . | 0 | . |  |  |
| *Blethisa multipunctata* | 1 | . | 0 | . |  |  |
| *Bembidion guttula* | 6 | . | 0 | . |  |  |
| *Bembidion mannerheimii* | 21 | 2 ± 2 | 0 | 0 ± 0 | -7.029 | < 0.001 |
| *Bembidion pygmaeum* | 1 | . | 0 | . |  |  |
| *Bembidion quadrimaculatum* | 0 | . | 1 | . |  |  |
| *Calathus fuscipes* | 15 | . | 0 | . |  |  |
| *Calathus rotundicollis* | 3 | . | 0 | . |  |  |
| *Carabus nemoralis* | 13 | 1 ± 1 | 44 | 4 ± 2 | 2.706 | 0.024 |
| *Harpalus autumnalis* | 1 | . | 0 | . |  |  |
| *Harpalus laevipes* | 0 | . | 1 | . |  |  |
| *Harpalus latus* | 1 | . | 14 | . |  |  |
| *Harpalus rubripes* | 2 | . | 6 | . |  |  |
| *Harpalus rufipalpis* | 4 | . | 1 | . |  |  |
| *Harpalus serripes* | 1 | . | 0 | . |  |  |
| *Harpalus signaticornis* | 2 | . | 0 | . |  |  |
| *Harpalus smaragdinus* | 4 | . | 4 | . |  |  |
| *Harpalus solitarsis** | 1 | . | 2 | . |  |  |
| *Harpalus tardus* | 5 | 1 ± 0 | 30 | 3 ± 1 | 2.865 | 0.019 |
| *Harpalus xanthopus* | 0 | . | 5 | . |  |  |
| *Lebia chlorocephala** | 1 | . | 0 | . |  |  |
| *Leistus ferrugineus* | 1 | . | 1 | . |  |  |
| *Leistus rufomarginatus* | 1 | . | 7 | . |  |  |
| *Licinus depressus* | 0 | . | 1 | . |  |  |
| *Loricera pilicornis* | 28 | 3 ± 2 | 1 | 0 ± 0 | -2.053 | 0.070 |
| *Nebria brevicollis* | 210 | 21 ± 13 | 29 | 3 ± 2 | -4.966 | < 0.001 |
| *Notiophilus aquaticus* | 1 | . | 1 | . |  |  |
| *Notiophilus biguttatus* | 4 | . | 12 | . |  |  |
| *Notiophilus germinyi** | 2 | . | 2 | . |  |  |
| *Notiophilus palustris* | 10 | . | 4 | . |  |  |
| *Notiophilus rufipes** | 4 | . | 1 | . |  |  |
| *Panagaeus bipustulatus* | 2 | . | 6 | . |  |  |
| *Patrobus atrorufus* | 0 | . | 2 | . |  |  |
| *Poecilus lepidus* | 1 | . | 0 | . |  |  |
| *Poecilus versicolor* | 17 | . | 0 | . |  |  |
| *Pseudoophonus rufipes* | 1 | . | 1 | . |  |  |
| *Pterostichus niger* | 9 | . | 0 | . |  |  |
| *Pterostichus nigrita* | 3 | . | 0 | . |  |  |
| *Pterostichus oblongopunctatus* | 16 | . | 0 | . |  |  |
| *Pterostichus rhaeticus* | 4 | . | 0 | . |  |  |
| *Pterostichus strenuus* | 22 | 2 ± 1 | 1 | 0 ± 0 | -11.202 | < 0.001 |
| *Stomis pumicatus* | 0 | . | 3 | . |  |  |
| *Syntomus truncatellus* | 0 | . | 1 | . |  |  |
| *Trechus obtusus* | 3 | . | 1 | . |  |  |
|  |  |  |  |  |  |  |
| **Spiders** |  |  |  |  |  |  |
| *Abacoproeces saltuum* | 1 | 0 ± 0 | 21 | 2 ± 1 | 2.350 | 0.043 |
| *Agraecina striata** | 2 | . | 0 | . |  |  |
| *Agroeca brunnea* | 5 | . | 2 | . |  |  |
| *Agroeca cuprea** | 2 | . | 1 | . |  |  |
| *Alopecosa pulverulenta* | 5 | . | 1 | . |  |  |
| *Anyphaena accentuata* | 0 | . | 8 | . |  |  |
| *Asthenargus paganus* | 1 | . | 0 | . |  |  |
| *Callilepis nocturna** | 2 | . | 0 | . |  |  |
| *Centromerus pabulator* | 1 | . | 1 | . |  |  |
| *Centromerus sylvaticus* | 6 | . | 1 | . |  |  |
| *Ceratinella brevis* | 7 | . | 5 | . |  |  |
| *Cheiracanthium virescens* | 1 | . | 0 | . |  |  |
| *Clubiona comta* | 1 | . | 5 | . |  |  |
| *Clubiona lutescens* | 5 | . | 4 | . |  |  |
| *Clubiona reclusa* | 2 | . | 0 | . |  |  |
| *Clubiona terrestris* | 4 | . | 6 | . |  |  |
| *Clubiona trivialis* | 1 | . | 0 | . |  |  |
| *Diaea dorsata* | 0 | . | 1 | . |  |  |
| *Dicymbium brevisetosum* | 4 | . | 3 | . |  |  |
| *Dicymbium tibiale* | 2 | . | 3 | . |  |  |
| *Diplocephalus latifrons* | 6 | 1 ± 0 | 76 | 8 ± 4 | 6.147 | < 0.001 |
| *Diplocephalus picinus* | 22 | 2 ± 2 | 29 | 3 ± 1 | 0.834 | 0.426 |
| *Dipostyla concolor* | 5 | . | 9 | . |  |  |
| *Dismodicus bifrons* | 1 | . | 4 | . |  |  |
| *Drassodes lapidosus* | 3 | . | 3 | . |  |  |
| *Drassyllus lutetianus* | 1 | . | 0 | . |  |  |
| *Drassyllus praeficus** | 10 | . | 0 | . |  |  |
| *Drassylus pusillus* | 1 | . | 0 | . |  |  |
| *Dysdera erythrina** | 6 | . | 3 | . |  |  |
| *Enoplognatha ovata* | 0 | . | 4 | . |  |  |
| *Enoplognatha thoracica* | 15 | . | 3 | . |  |  |
| *Episinus angulatus* | 1 | . | 14 | . |  |  |
| *Episinus truncatus** | 1 | . | 0 | . |  |  |
| *Erigone atra* | 2 | . | 2 | . |  |  |
| *Erigone dentipalpis* | 1 | . | 2 | . |  |  |
| *Erigonella hiemalis* | 0 | . | 1 | . |  |  |
| *Ero furcata* | 0 | . | 1 | . |  |  |
| *Euophrys frontalis* | 4 | . | 4 | . |  |  |
| *Euryopis flavomaculata* | 41 | 4 ± 2 | 62 | 6 ± 2 | 1.084 | 0.307 |
| *Gongylidium rufipes* | 0 | . | 2 | . |  |  |
| *Haplodrassus signifer* | 4 | . | 2 | . |  |  |
| *Haplodrassus silvestris* | 3 | . | 2 | . |  |  |
| *Haplodrassus umbratilis* | 34 | 3 ± 3 | 10 | 1 ± 1 | -3.589 | 0.006 |
| *Harpactea rubicunda* | 25 | 3 ± 1 | 9 | 1 ± 0 | -2.530 | 0.032 |
| *Heliophanus flavipes* | 0 | . | 1 | . |  |  |
| *Linyphia hortensis* | 0 | . | 5 | . |  |  |
| *Linyphia triangularis* | 0 | . | 3 | . |  |  |
| *Malthonica pagana* | 2 | . | 0 | . |  |  |
| *Maso sundevalli* | 4 | . | 0 | . |  |  |
| *Meioneta rurestris* | 7 | . | 0 | . |  |  |
| *Meioneta saxatilis* | 2 | . | 5 | . |  |  |
| *Mermessus trilobatus* | 1 | . | 0 | . |  |  |
| *Micaria fulgens* | 9 | . | 1 | . |  |  |
| *Micaria pulicaria* | 1 | . | 0 | . |  |  |
| *Microneta viaria* | 2 | . | 1 | . |  |  |
| *Mioxena blanda** | 1 | . | 0 | . |  |  |
| *Neottiura bimaculata* | 0 | . | 3 | . |  |  |
| *Neriene clathrata* | 1 | . | 0 | . |  |  |
| *Oedothorax retusus* | 3 | . | 0 | . |  |  |
| *Ozyptila praticola* | 168 | 17 ± 6 | 435 | 44 ± 7 | 2.845 | 0.019 |
| *Ozyptila scabricola** | 1 | . | 0 | . |  |  |
| *Ozyptila trux* | 4 | . | 2 | . |  |  |
| *Pachygnatha degeeri* | 15 | . | 2 | . |  |  |
| *Palliduphantes pallidus* | 1 | . | 6 | . |  |  |
| *Pardosa amentata* | 0 | . | 2 | . |  |  |
| *Pardosa lugubris* | 1664 | 166 ± 50 | 1968 | 197 ± 70 | 0.385 | 0.709 |
| *Pardosa palustris* | 1 | . | 2 | . |  |  |
| *Pardosa prativaga* | 15 | 2 ± 1 | 5 | 1 ± 0 | -1.979 | 0.079 |
| *Philodromus aureolus* | 0 | . | 2 | . |  |  |
| *Phrurolithus festivus* | 29 | 3 ± 1 | 4 | 0 ± 0 | -3.170 | 0.011 |
| *Piratula hygrophilus* | 33 | 3 ± 3 | 1 | 0 ± 0 | -1.933 | 0.085 |
| *Piratula latitans* | 3 | . | 1 | . |  |  |
| *Pisaura mirabilis* | 2 | . | 1 | . |  |  |
| *Pocadicnemis pumila* | 0 | . | 1 | . |  |  |
| *Poeciloneta variegata* | 5 | . | 2 | . |  |  |
| *Robertus lividus* | 5 | . | 1 | . |  |  |
| *Saaristoa abnormis* | 0 | . | 1 | . |  |  |
| *Tegenaria domestica* | 2 | . | 0 | . |  |  |
| *Tenuiphantes flavipes* | 171 | 17 ± 6 | 142 | 14 ± 7 | -0.635 | 0.541 |
| *Tenuiphantes mengei* | 9 | . | 2 | . |  |  |
| *Tenuiphantes tenebricola* | 1 | . | 1 | . |  |  |
| *Tenuiphantes tenuis* | 15 | 2 ± 1 | 47 | 5 ± 4 | 2.714 | 0.024 |
| *Textrix denticulata* | 0 | . | 1 | . |  |  |
| *Thanatus sabulosus** | 1 | . | 0 | . |  |  |
| *Tiso vagans* | 21 | 2 ± 2 | 0 | 0 ± 0 | -7.305 | <0.001 |
| *Trochosa ruricola* | 23 | 2 ± 1 | 1 | 0 ± 0 | -2.404 | 0.040 |
| *Trochosa terricola* | 13 | 1 ± 1 | 8 | 1 ± 0 | -0.866 | 0.409 |
| *Troxochrus scabriculus* | 15 | 2 ± 1 | 27 | 3 ± 2 | 1.070 | 0.312 |
| *Walckenaeria acuminata* | 0 | . | 2 | . |  |  |
| *Walckenaeria antica* | 1 | . | 0 | . |  |  |
| *Walckenaeria atrotibialis* | 15 | 2 ± 1 | 31 | 3 ± 2 | 1.928 | 0.086 |
| *Walckenaeria cucullata* | 0 | . | 1 | . |  |  |
| *Xerolycosa miniata* | 2 | . | 0 | . |  |  |
| *Xerolycosa nemoralis* | 3 | . | 0 | . |  |  |
| *Xysticus cristatus* | 0 | . | 1 | . |  |  |
| *Zelotes electus* | 1 | . | 1 | . |  |  |
| *Zelotes subterraneus* | 33 | 3 ± 1 | 4 | 0 ± 0 | -3.614 | 0.006 |
| *Zilla diodia* | 1 | . | 0 | . |  |  |
| *Zodarion rubidum* | 1 | . | 1 | . |  |  |
| *Zora spinimana* | 33 | 3 ± 1 | 5 | 1 ± 0 | -3.264 | 0.010 |
